# Mechanical Properties of FDM Printed PLA Parts before and after Thermal Treatment

**DOI:** 10.3390/polym13081239

**Published:** 2021-04-11

**Authors:** Ali Chalgham, Andrea Ehrmann, Inge Wickenkamp

**Affiliations:** 1 Faculty of Engineering and Mathematics, Bielefeld University of Applied Sciences, 33619 Bielefeld, Germany; ali.chalgham@gmail.com (A.C.); andrea.ehrmann@fh-bielefeld.de (A.E.); 2 Mechanical Department, Ecole Nationale d’Ingénieurs de Sfax (ENIS), Sfax 3038, Tunisia

**Keywords:** polylactic acid, heat treatment, mechanical properties, printing parameters, finger orthosis

## Abstract

Fused deposition modeling (FDM) is one of the most often-used technologies in additive manufacturing. Several materials are used with this technology, such as poly(lactic acid) (PLA), which is most commonly applied. The mechanical properties of 3D-printed parts depend on the process parameters. This is why, in this study, three-point bending tests were carried out to characterize the influence of build orientation, layer thickness, printing temperature and printing speed on the mechanical properties of PLA samples. Not only the process parameters may affect the mechanical properties, but heat after-treatment also has an influence on them. For this reason, additional samples were printed with optimal process parameters and characterized after pure heat treatment as well as after deformation at a temperature above the glass transition temperature, cooling with applied deformation, and subsequent recovery under heat treatment. These findings are planned to be used in a future study on finger orthoses that could either be printed according to shape or in a flat shape and afterwards heated and bent around the finger.

## 1. Introduction

3D printing belongs to the emerging technologies of our time. Besides prototyping, diverse 3D printing technologies can be used for a broad range of applications, such as sensors [1,2,3], microfluidic and MEMS devices [4,5,6], biotechnology [7,8], or workwear and protective textiles [9,10].

Amongst the diverse techniques available currently, the fused deposition modeling (FDM) technology belongs to the most often-used ones, since it is easily available at low cost [11]. Amongst the broad range of materials available for FDM printers, poly(lactic acid) (PLA) still is the most-often used one due to its ease of use. Another important property of this bio-based material is its biocompatibility and biodegradability, making it useful for tissue engineering or bone repair [12,13,14], but also for medical aids [15,16].

For all these possible applications, it is necessary to optimize the mechanical properties and sometimes also the surface roughness and waviness of the 3D-printed parts [17,18,19,20]. One possibility to increase the elastic modulus or the tensile stress and other mechanical properties is by integrating continuous filaments into the polymer feedstock [21,22]. Alternatively, short fibers or nanoparticles can be used to improve mechanical and morphological properties [23,24,25]. However, such composite printed objects are not suitable for bone-repair applications as the integrated materials are usually not biocompatible or even biodegradable.

Another interesting property of PLA is its shape memory, meaning that a PLA 3D printed object can be deformed, ideally at a temperature above the glass transition temperature, and the original shape is automatically restored when the object is heated again to a temperature of about 60–90 °C [26,27,28]. However, this works only as long as no connections are broken, leading in reality to the necessity to construct such objects carefully to avoid breaks and thus to optimize regeneration properties [29,30]. Other FDM printing materials may also show shape-memory properties, such as polyurethane or poly(vinyl alcohol) (PVA) [31,32], but they are less common in the FDM process.

Besides this possibility to restore the original shape after a deformation, it must be mentioned that PLA can also be actively deformed at the aforementioned temperatures, enabling using a well-printable shape during printing, after which the part is bent into the final shape. This process is often called 4D printing [33,34]. Especially for orthoses, this process can be highly interesting since it not only allows for printing an easier and often more stable shape with an optimal orientation of the strongest directions in the final orthosis with highly anisotropic mechanical properties; it may also be used to tailor the shape individually to each patient. This is why we report here on the influence of different heat treatments, which would be necessary to model the shape of 3D-printed orthoses, on the mechanical properties of 3D-printed objects. In addition, we depict the impact of printing orientation and other printing parameters on the flexural strength of 3D-printed specimens.

## 2. Materials and Methods

3D printing was performed on a Raise 3D Pro2 Plus printer (Raise3D, Shanghai, China) with a nozzle diameter of 0.4 mm, using PLA filament with a diameter of 1.75 mm (FilamentWorld, Neu-Ulm, Germany). Table 1 depicts the standard parameters as well as the variations tested in this study.

Samples for 3-point bending tests were printed in dimensions of 80 mm × 10 mm × 4 mm, according to ISO 178:2019 (Plastics—Determination of flexural properties). For each parameter set, 3 specimens were printed.

A Sauter TVM-N universal testing machine (Kern & Sohn GmbH, Balingen-Frommern, Germany) was applied for 3-point bending tests, using a speed of (5 ± 1) mm/min according to ISO 178:2019.

The design of the finger orthosis used to test shaping under heat treatment and reshaping was taken from [35].

## 3. Results and Discussion

**Figure 1:**
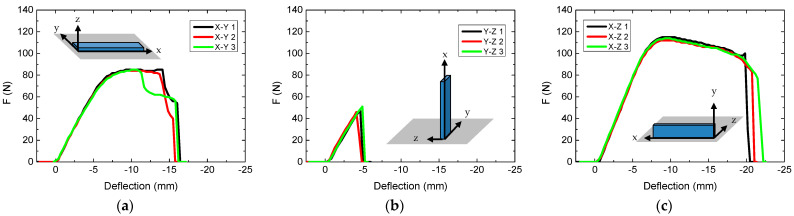
Force-deflection curves, obtained from 3-point bending tests for various build orientations: (**a**) in the x–y plane (i.e., flat on the large side); (**b**) in the y–z plane (i.e., on the smallest side); (**c**) in the x–z plane (i.e., on the long, thin side).

First, the build orientation was varied. Flat samples are normally printed with the largest side oriented in the x–y plane of the printer. The results of modifications of the build orientation are depicted in Figure 1, showing tests of all three specimens per samples. 

Comparing the three nominally identical specimens per sample, the force-deflection curves are quite similar for each build orientation. This shows that printing more samples is not necessary since no large deviations can be expected. Only in the case of the x–y plane (Figure 1a), one of the samples shows a behavior different from the others, which can be attributed to fully breaking, while the other specimens were not completely separated.

While printing the specimen in the y–z orientation (i.e., on the smallest side of the samples) resulted in the lowest forces at break (Figure 1b), the largest forces were necessary in the x–z plane, that is, when the samples were placed on the long, thin side instead of the intuitively often chosen largest side (Figure 1c). Due to this result and the desired optimization with respect to the breaking force, the following investigations were performed with the specimens printed on the long side, that is, in the x–z plane. It should be mentioned that even the printing time is slightly reduced for this direction (14.4 min), as compared to the x–y direction (15.4 min), while printing in the y–z plane took nearly twice as long (28.6 min). Figure 1c is thus the reference for all following tests. 

**Figure 2:**
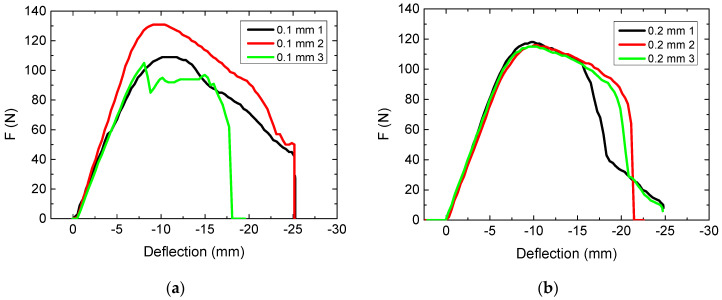
Force-deflection curves, measured in the 3-point bending tests for varying layer thicknesses: (**a**) 0.1 mm; (**b**) 0.2 mm.

Next, the dependence of the mechanical properties on the layer thickness was tested. The results are depicted in Figure 2. 

The influences of the layer thickness on the mechanical properties of 3D-printed specimens reported in the literature differ, suggesting that this effect is not independent from printer and printing material. Ayrilmis et al., for example, found an increase of tensile and bending properties of PLA/wood printed specimens for a decreasing layer thickness [36]. Similarly, Yao et al. found an increase of the tensile failure strength of PLA samples with decreasing layer thickness [37]. On the other hand, García Plaza et al. found no clear effect of the layer thickness on dimensional and mechanical properties of PLA specimens [38]. For ABS, Sood et al. found the opposite behavior, increased mechanical properties for an increased layer thickness [39]. Song et al., to show another possible effect, found an average layer height of 0.2 mm to be optimal for PLA, testing layer thicknesses from 0.1 mm to 0.4 mm [40].

Here, results for layer thicknesses of 0.2 mm (Figure 2b) and 0.3 mm (Figure 1c) are quite similar, while for a layer thickness of 0.1 mm, the three specimens show strongly different force-deflection curves (Figure 2a). This suggests using 0.2 mm or 0.3 mm as the layer thickness to receive reliable bending rigidity. Combined with the reduced printing time for a layer thickness of 0.3 mm (14.4 min, as compared to 20.7 min for layer thickness 0.2 mm and 38.3 min for layer thickness 0.1 mm), this value of 0.3 mm was kept for the next tests.

**Figure 3:**
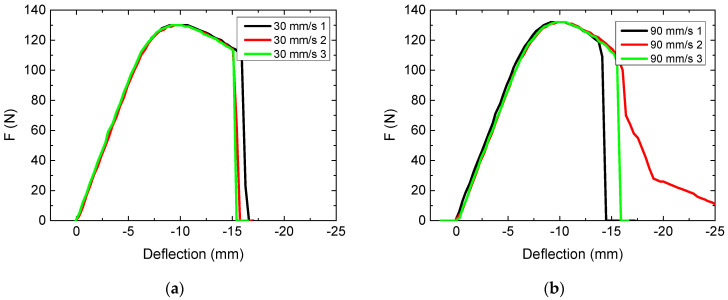
Force-deflection curves, obtained from 3-point bending tests for various printing speeds: (**a**) 30 mm/s; (**b**) 90 mm/s.

The printing speed was varied as the next parameter. Figure 3 depicts the results of these tests. 

Interestingly, both maxima are reached at slightly higher forces than measured for the average printing speed of 60 mm/s (Figure 1c). Possible reasons for this finding are different environmental conditions, such as temperature or relative humidity, which may influence the printing or even the testing process. Samples were thus printed again with a printing speed of 60 mm/s after cleaning the nozzle and at identical environmental conditions, but showing exactly the same maximum force of (114 ± 1) N as in the previous test, where a maximum force of (114 ± 2) N was found. This verifies that the intermediate printing speed leads indeed to significantly smaller maximum forces and suggests a larger test series with more values of the printing speed. 

The printing duration varies naturally with the printing speed, from 13.5 min (for 90 mm/s) to the already known value of 14.4 min (60 mm/s) to even 18.9 min (for 30 mm/s). This implies using the maximum speed of 90 mm/s. However, only in this case (Figure 3b), strong variations of the deflection at break are visible, suggesting to use a slightly lower printing speed.

**Figure 4:**
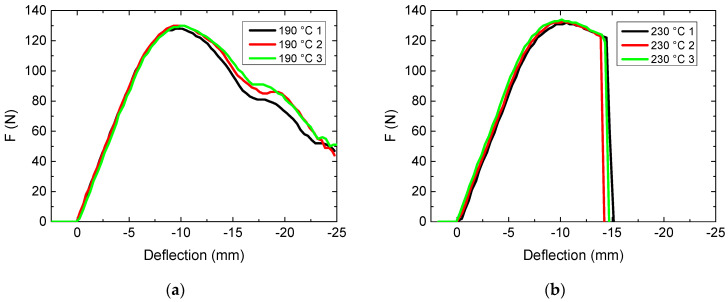
Force-deflection curves, obtained from 3-point bending tests for various nozzle temperatures: (**a**) 190 °C; (**b**) 230 °C.

For the sake of comparability, the evaluation of the impact of the printing temperature (Figure 4) was thus performed using a printing speed of 60 mm/s again.

Here, a clear qualitative difference between the different printing temperatures is visible. While samples printed at a temperature of 230 °C (Figure 4b) behave qualitatively similar to those printed at 210 °C (Figure 1c), the lower printing temperature of 190 °C can apparently be used to avoid breaking of the samples—a behavior that is strongly advantageous in the case of orthoses where breaking completely may mean that the patient is hurt by the broken parts.

On the other hand, comparing the maximum forces, there are slightly higher values found for 230 °C ((133 ± 1) N) than for the printing temperature of 190 °C ((129 ± 1) N). These differences are, however, in the range of only 3% and can thus be regarded as less important than avoiding full separation of sample parts during breaking.

It must be mentioned that both maximum forces are again significantly higher than the value of ((114 ± 1) N) found for the intermediate temperature. Apparently, the impact of the nozzle temperature also should be investigated in more detail in future tests. These unexpected findings, showing that average values are not necessarily the optimum but can even be the worst choice, can on the one hand explain the contradictory findings reported in the literature regarding the influence of printing parameters such as the layer thickness on the mechanical properties of 3D-printed samples. On the other hand, a similar effect was found for the heat-triggered shape regeneration of 3D-printed objects after deformation, where an intermediate infill degree was also found to result in lower regeneration capability than higher or lower infill degrees [29,30].

**Figure 5:**
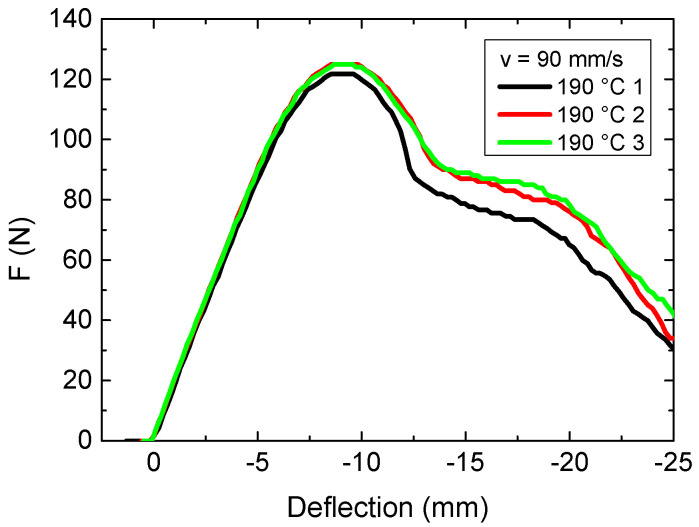
Force-deflection curves, obtained from 3-point bending tests for a nozzle temperature of 190 °C, a velocity of 90 mm/s and an x–z orientation.

From these experiments, the following optimum printing parameters can be defined: printing in the x–z plane at 0.3 mm layer height with a nozzle temperature of 190 °C and an intermediate printing speed of 60 mm/s. Next, it was investigated whether using a nozzle temperature of 190 °C combined with a higher printing temperature speed of 90 mm/s could also help to avoid breaking of the samples and at the same time increase the production speed, as compared to the printing speed of 60 mm/s, as depicted in Figure 4a. Figure 5 thus shows force-deflection curves of samples printed with this parameter combination.

In comparison with the samples printed with a velocity of 60 mm/s, not much difference is visible. This is why the next samples were again printed with a velocity of 90 mm/s.

**Figure 6:**
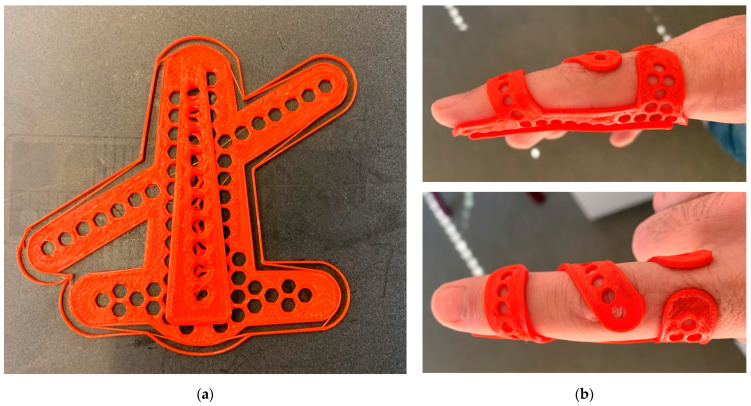
3D-printed finger orthosis (**a**) on the printing bed after printing; (**b**) after bending around a finger.

The final aim of this project is printing finger orthoses, as depicted in Figure 6, ideally in the way shown here, that is, by printing them flat, followed by heating them to a temperature above the glass transition temperature of approx. 60 °C and bending them around the finger of the patient.

**Figure 7:**
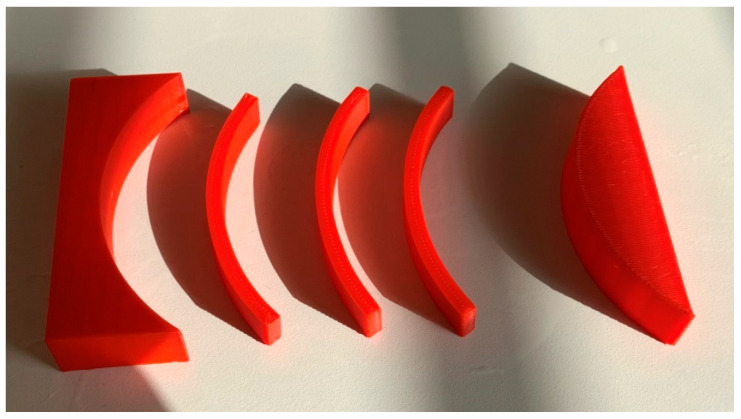
Preparation of samples bent at high temperature with both parts of the mold defining the bent shape (left and right).

**Figure 8:**
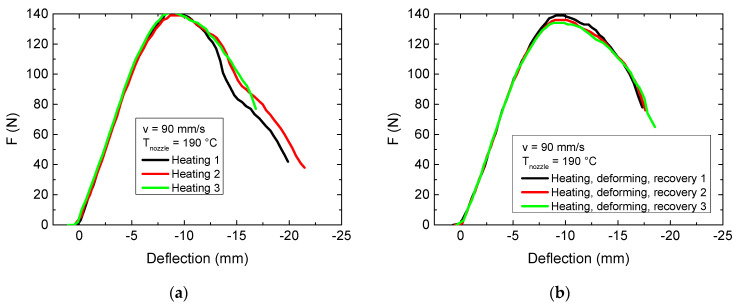
Force-deflection curves, obtained from 3-point bending tests after different heat post-treatments: (**a**) pure heat treatment without mechanical bending; (**b**) bending and relaxation under heat treatment.

This means on the one hand that the optimum printing parameter “in the x–z plane” cannot be fulfilled. On the other hand, it is necessary to investigate also the mechanical properties after such a heat treatment. In addition, it must be investigated whether bending the samples at a temperature slightly above the glass transition temperature to produce an individualized orthosis reduces its mechanical properties. This test was performed by heating the samples in a water bath to a temperature of 75 °C, that is, well above the glass transition temperature of PLA, for two minutes and deforming it in a mold with a bending radius of 100 mm (cf. Figure 7). After letting a sample cool down inside the mold, it was again inserted in water of 75 °C to let it recover its original shape due to its shape-memory properties [29,30]. To test the pure effect of a temperature treatment, another set of samples was also heated in a water bath of temperature 75 °C for 2 min, but this time without any mechanical impact. The results of these experiments are depicted in Figure 8.

On the one hand, Figure 8a shows clearly that heating the sample is supportive for the maximum force. On the other hand, this maximum force is not significantly reduced by one deforming and recovery cycle (Figure 8b). This suggests that the preparation of finger orthoses and similar orthoses is possible by 3D printing a flat shape, followed by bending it to fit it to a proband’s finger. Instead, the whole curve becomes slightly broader, meaning that larger bending is possible without a break of the sample, which is even advantageous for the application of 3D-printed orthoses. Studies of other groups showed for some 3D printing materials that either forced cooling or temperature post-treatment could be used to increase maximum force and displacement at break [41,42,43]; however, tests of mechanical properties after deformation and recovery of the original shape usually show slightly reduced mechanical properties [44].

It must be mentioned, however, that the tests performed here used a relatively large bending radius in connection with a relatively thin sample. Future tests must investigate which ratios of orthosis thickness and bending radius can be used without a significant decrease of the maximum force or other important mechanical properties.

It should also be mentioned that PLA is not the only shape-memory polymer that can be 3D-printed [31,32] and that it can be assumed that polyurethane or PVA could be used similarly. However, polyurethane shows two glass transition temperatures, the higher of which is—depending on the exact composition—higher than that of PLA, making it more complicated to fit an orthosis of this material to the human body, while PVA is water-soluble and thus not suitable to serve as an orthosis.

## 4. Conclusions

PLA samples were 3D-printed using the FDM method, and the influences of different printing parameters on the mechanical properties as measured in a 3-point bending test were investigated. The best of the tested parameters were an x–z printing orientation (i.e., on a long edge), a nozzle temperature of 190 °C to avoid breaking of the samples, a printing speed of 90 mm/s and a layer thickness of 0.3 mm.

Samples printed with these parameters were subjected to heat post-treatment at 75 °C, leading to a slightly increased maximum force at bending. Finally it was found that deforming and recovery under heat treatment does not reduce the maximum forces significantly. This enables 3D printing of orthoses in a flat state and afterwards bending them to fit the desired part of the human body.

Future research will concentrate on finding the borders of this method in terms of structure thickness and bending radius.

## Figures and Tables

**Table 1:** Printing parameters–standard values and variations.

Parameter	Standard Value	Variations
Nozzle temperature/°C	210	190, 230
Heating bed temperature/°C	60	-
Infill density/%	30	-
Number of shells	4	-
Infill pattern	Lines	-
Raster angle	0°/90°	-
Layer thickness/mm	0.3	0.1, 0.2
Printing speed/(mm/s)	60	30, 90
Build orientation	x-z plane	y-z plane, x-y plane

## Data Availability

Data are contained within the article.
